# HPV & head and neck cancer: a descriptive update

**DOI:** 10.1186/1758-3284-1-36

**Published:** 2009-10-14

**Authors:** Peter KC Goon, Margaret A Stanley, Jörg Ebmeyer, Lars Steinsträsser, Tahwinder Upile, Waseem Jerjes, Manuel Bernal-Sprekelsen, Martin Görner, Holger H Sudhoff

**Affiliations:** 1 Department of Pathology, University of Cambridge, Cambridge, UK; 2 Department of Otolaryngology, Head and Neck Surgery, Bielefeld Academic Teaching Hospital, Bielefeld, Germany; 3 Department of Plastic Surgery and Burn Centre, BG University Hospital Bergmannsheil, Ruhr University Bochum, Germany; 4 The University College London, Ear Institute, Gray's Inn Road, London, UK; 5 Department of Otolaryngology, Head and Neck Surgery, Hospital Clinic of Barcelona, University of Barcelona, Spain; 6 Department of Hematology and Oncology, Bielefeld Academic Teaching Hospital, Bielefeld, Germany

## Abstract

The incidence of head and neck squamous cell carcinoma (HNSCC) has been gradually increasing over the last three decades. Recent data have now attributed a viral aetiology to a subset of head and neck cancers. Several studies indicate that oral human papillomavirus (HPV) infection is likely to be sexually acquired. The dominance of HPV 16 in HPV+ HNSCC is even greater than that seen in cervical carcinoma of total worldwide cases. Strong evidence suggests that HPV+ status is an important prognostic factor associated with a favourable outcome in head and neck cancers.

Approximately 30 to 40% of HNSCC patients with present with early stage I/II disease. These patients are treated with curative intent using single modality treatments either radiation or surgery alone. A non-operative approach is favored for patients in which surgery followed by either radiation alone or radiochemotherapy may lead to severe functional impairment. Cetuximab, a humanized mouse anti-EGFR IgG1 monoclonal antibody, improved locoregional control and overall survival in combination with radiotherapy in locally advanced tumours but at the cost of some increased cardiac morbidity and mortality.

Finally, the improved prognosis and treatment responses to chemotherapy and radiotherapy by HPV+ tumours may suggest that HPV status detection is required to better plan and individualize patient treatment regimes.

## Introduction and epidemiology

Cancer of the head and neck, includes all cancers arising from the upper aerodigestive tract, and typically refers to squamous cell carcinomas of the head and neck, which are the predominant group. The incidence of head and neck squamous cell carcinoma (HNSCC) has been gradually increasing over the last 3 decades. It is the 5^th ^leading cause of cancer by incidence and the 6^th ^leading cause of cancer mortality in the world. In 2002, approximately 405,000 were reported and 211,000 deaths occurred worldwide [1]. It is likely that approximately 500,000 cases worldwide will arise this year, and that only 40-50% will survive for 5 years [2-4]. The typical case presents late, as advanced metastatic cancer, and accounts for the poor mortality rate. The most important risk factors so far identified are tobacco and alcohol. These appear to have a synergistic effect on the mucosal surfaces. Carcinogens produced by tobacco such as nitrosamines and benz-(a)-pyrene, produce the types of guanine nucleotide changes found in the p53 mutations seen in HNSCC. Alcohol effects are less clear, but may act by being metabolized to acetaldehyde, which damages DNA and traps glutathione, which is an important peptide in detoxification of carcinogens. Furthermore, alcohol can also induce cytochrome p450 enzyme CYP2E1, which is involved in inactivation of various pro-carcinogens found in alcoholic drinks and tobacco smoke [5-8]. Indeed, one study has found a 20-fold increased risk of oral and pharyngeal cancer below age 46 for heavy smokers, and a 5-fold increase for heavy drinkers; if there was both heavy drinking and smoking, this combination led to an almost 50-fold increase in risk [9].

Cannabis has been postulated to have a role in the pathogenesis of HNSCC. Smoking cannabis theoretically has the potential for a contributory role in HNSCC as qualitatively, cannabis smoke is fairly similar to tobacco smoke but actually has a greater concentration of the aromatic poly-carbon carcinogens found in cigarette smoke. Furthermore, cannabis is usually smoked unfiltered, allowing a greater concentration of toxin unfettered access to the mucosal surfaces. However, the vast majority of cannabis smokers do not smoke anywhere near the intensity and numbers that ordinary cigarette smokers do on a regular basis. Only one study of note has so far shown a statistically significant increased risk of head and neck cancer [10]. Further careful case-control studies aimed at elucidating this connection have not demonstrated the same result. Nearly all of the studies have limitations of small sample size and insufficient statistical power. At present, the evidence for this postulated risk factor remains equivocal. If there is a link, it appears to be a small one; and it will take a large multi-centre study to achieve the statistical power to demonstrate this.

Diet is another factor implicated in the aetiology of HNSCC, especially a low consumption of fibre and vitamins in the form of fresh fruit and vegetables [11]. Oral hygiene and the state of dentition have also been linked to an increased risk of developing oro-pharyngeal cancer. More than 20 teeth lost, more than 5 defective teeth and poorly fitting or defective complete dentures were significantly associated with an increased risk [12]. These are difficult factors to separate from high alcohol and tobacco consumption, as deprived low-income communities traditionally have high consumption of the main risk factors and low consumption of a better diet, poor dentition and oral hygiene.

### Human papillomavirus (HPV) as a risk factor

Recent data have now attributed a viral aetiology to a subset of head and neck cancers; the human papillomavirus (HPV). The first clue to this was suggested in 1983 by a Finnish team led by Stina Syrjänen, who noted that 40% of the cancers in their study contained histological and morphological similarities with HPV-associated lesions [13]. Since then, growing epidemiological evidence strongly support a role for HPV in a subset of HNSCC. For instance, the incidence of HNSCC in the USA has reached a plateau while strong anti-smoking campaigns have resulted in a decrease in the number of current smokers from 42.5% in 1965 to 20.9% in 2005, while the number of never smokers has risen from 44.0% in 1965 to 57.5% in 2005. Although certain subsets of HNSCC have fallen in parallel with the reduction in smoking, rates of oropharyngeal cancers, particularly tongue and tonsillar cancers, have risen steadily by 2.1% and 3.9% among men and women respectively, aged 20-44 years from 1973 to 2001 [14,15]. Similar patterns have been noted in Stockholm in Sweden, for tonsillar cancer which rose 2.8-fold between 1970 and 2002, with a concurrent rise in HPV+ tonsillar cancer, which rose by 2.9-fold during that time, despite a decreasing prevalence in smoking [16]. A similar trend has also been noted in Finland [17].

Several studies indicate that oral HPV infection is likely to be sexually acquired. D'Souza and colleagues recently showed in a case-control study [18], that a high (26 or more) number of lifetime vaginal-sex partners and 6 or more lifetime oral-sex partners were associated with an increased risk of HNSCC (OR 3.1 and 3.4 respectively). An increased risk of HPV-associated HNSCC in female patients with a history of HPV-associated anogenital cancers and their male partners is also consistent with HPV transmission to the oropharyngeal cavity [19]. Serological and molecular markers of HPV infection are also associated with increased risks of HNSCC. Hansson et al [20] found a strong association between the detection of high-risk HPV DNA in the oral cavity and oro-pharyngeal carcinoma (OR 230; CI 45-1200) after adjusting for alcohol and tobacco usage. A separate, nested case-control study demonstrated an increased risk of greater than 2-fold for subsequently developing oral cancer if there was HPV-16 seropositivity [21].

Establishing the evidence for a causal role for HPV in a certain subset of HNSCC has been compounded by the heterogeneity of the tumours and multiple primary anatomical sites. Since Syrjanen's observations in 1985, there have been numerous publications studying HPV DNA detection in HNSCC with rates varying from 0% to 100% of tumours studied. These studies employed different detection techniques such as lower sensitivity methods i.e. in situ hybridisation, Southern Blot hybridisation or high sensitivity methods such as PCR; different sampling methods such as biopsies, scrapes, brushes, oral rinses, etc; different storage procedures such as fresh frozen or formalin fixed paraffin embedded samples. It is clear that that the non-standardisation of these multiple variables has not brought clarity to the field. Kreimer et al in 2005 conducted a systematic meta-analysis to review the available literature in the field and ascertain the worldwide prevalence of HPV in HNSCC [22]. They analysed 60 eligible studies using PCR detection from 26 countries which included 5046 cases of squamous cell cancers; 2642 oral cancers, 969 oropharyngeal cancers and 1435 laryngeal cancers. HPV prevalence was 35.6% in oropharyngeal cancers, 23.5% in oral cancers and 24.0% in laryngeal cancers. Overall prevalence of HPV in HNSCC was estimated at 26%. HPV 16 was by far the commonest subtype in all types of HPV+ cancers; 86.7% of oropharyngeal, 68.2% of oral and 69.2% of laryngeal cancers. HPV 18 was next most common but found in only 1% of oropharyngeal, 8.0% of oral and 3.9% of laryngeal cancers. A more recent meta-analysis by Termine et al 2008, estimated that from studies utilising only FFPE samples, the pooled prevalence of HPV detected in these HNSCC (defined as SCCs originating in the oral, pharyngeal and laryngeal cavities only) was 34.5% [23].

### Insights into the molecular mechanisms of HPV carcinogenesis

The dominance of HPV 16 in HPV+ HNSCC (85%-95%) is even greater than that seen in cervical carcinoma (approximately 50-60%) of total worldwide cases. This is likely to reflect a difference in life cycles of the different HPV subtypes in different mucosal locations, with an associated difference in mucosal immune responses. The high-risk subtypes of HPV involved in cervical carcinogenesis have been defined [24]. Meta-analyses have shown that the HPV subtypes associated with HNSCC are broadly similar (but not identical) with those seen in cervical carcinoma [22,23].

It has been shown that these subtypes (particularly 16) are able to transform and immortalise cells in vitro. These effects are predominantly due to the E6 and E7 oncogenes, which bind and enhance degradation of P53 and RB tumour suppressor genes respectively. There is evidence that immortalisation of oral keratinocytes and epithelial cells occur quite readily [25,26]. Furthermore, one study showed that HPV+ HNSCC had mutations in the promoter/enhancer region that conferred significantly increased activity and thus could increase the expression of E6 and E7 in these oral keratinocytes [27]. However, it is interesting to note that the classically low-risk HPV 6 and 11 subtypes have been found in some tonsillar and laryngeal carcinomas. It is well known that in the rare event of benign laryngeal papillomas undergoing malignant transformation, HPV 11 has been most commonly implicated. HPV 6 and 11 have also been implicated in malignancies such as Ackerman's tumour (verrucous carcinoma of the oral cavity). This is analogous to the finding of HPV 6 and 11 in Buschke-Lowenstein tumours. It is clear that in certain rare malignancies, HPV 6 and 11 can play a role.

**Integration **of HPV DNA into genomic DNA is a common event in cervical carcinoma, indeed, the dominant form. It has been shown that integration usually leads to disruption and/or deletion of HPV E1 or E2 open reading frame (ORF), which are important for viral replication and transcription. E2 functions also as a repressor of E6 and E7 and disruption of E2 activity allows increased E6 and E7 expression, thus maintaining the immortalised phenotype [28]. Integration of HPV 16 DNA also correlates with a selective growth advantage and may allow the cancerous cell to outgrow its rivals, this may be an important step in the pathway of oncogenesis [29]. However, despite the dominance of the integrated HPV genome in terms of cervical carcinogenesis, 15-30% of cervical cancer contain HPV only in the episomal form [30-32]. In some of these cases, investigators have found deletions in the YY1-binding sites of the LCR (long control region) of HPV 16 episomal DNA which may allow elevated activity of the E6/E7 promoter [33,34]. It is clear that the actual molecular pathway to cervical carcinogenesis is far from homogeneous.

The situation in head and neck cancers is less than clear but heterogeneity and the existence of multiple pathways to carcinogenesis is highly likely. Koskinen et al (2003) reported that in their series of head and neck cancers, 61% were HPV DNA positive. HPV 16 was the dominant subtype, and found in 84% of HPV+ cancers. RT-PCR analysis demonstrated that of the HPV 16+ samples, 48% were integrated, 35% were episomal and 17% were of both mixed episomal and integrated forms [35]. Tonsillar carcinomas have been reported to have the highest prevalence rate of HPV DNA contained within cancerous cells (51%) of all the forms of head and neck cancers [17]. Mellin et al 2002 reported that all 11 cases of HPV+ tonsillar carcinomas in their series contained HPVDNA in episomal form [36]. Another study in 1992 reported two HPV 16+ tonsillar carcinomas which contained episomal HPV DNA, and two HPV 33+ tonsillar carcinomas in which one was integrated and the other had mixed forms [37]. It is unclear why tonsillar carcinomas appear to have a higher predominance of episomal HPV DNA than other types of head and neck cancer. It is likely though, that these various observations suggest a high heterogeneity and variation in the oncogenic pathways among these tumours.

The **viral load **of HPV in head and neck cancers appear to vary considerably. Available data suggest that oral cavity HPV viral load measured by DNA is lower than in the cervix [38]. Tonsillar cancers appear to show a wide variation in HPV copy numbers. The study from Mellin et al 2002 detected 10 - 15,400 (median of 190) HPV 16 copies per beta-actin copy from 11 HPV 16+ tumours [36]. Of note was the fact that the 6 patients with > 190 HPV copies/Beta-actin had significantly better clinical outcome in terms of tumour free status and survival rate at the 3 year mark. The authors concluded that the data suggested that a higher viral load could be a favourable prognostic indicator. Klussmann et al showed in their study that in 7 HPV 16+ tonsillar carcinomas, the viral load ranged from 6.6 × 10-4 to 172.4 copies/Beta-globin copy in whole tissue. Laser microdissection of the samples showed that the HPV DNA was present in the dissected tumour cells and not in control tissue. Mean HPV 16 loads in dissected sections ranged from 6 - 150 copies/Beta-globin copy which was higher than in the corresponding whole tissue (thus demonstrating a dilution effect) [39]. Koskinen et al reported that the median copy number of E6 DNA was about 80,000 fold higher in tonsillar cancers as compared to non-tonsillar head and neck cancers. Furthermore, they reported that tumours with episomal DNA had larger tumours than patients with mixed or integrated forms of viral DNA [35]. This suggests that the higher copy number of episomal viral DNA was able to induce more rapid growth, perhaps by higher expression of the viral oncogenes. Weinberger et al (2006) demonstrated that HPV 16 viral load and p16 expression could be used to classify head and neck cancers into 3 distinct profiles: Class I, HPV-, p16 low; Class II, HPV+, p16 low; and Class III, HPV+, p16 high. Of note, was that Class III tumours had a significantly increased 5 year survival, increased disease-free survival rate and decreased local recurrence rate, compared to tumours in the other 2 classes. Median HPV viral load DNA (copies HPV 16/human genome) was 0.0 copies, 3.6 copies and 46.0 copies for Classes I, II and III respectively [40]. These results were consistent with those from Mellin et al (2002) [36].

### Clinical implications of HPV+ Head and neck cancers

Accumulating evidence suggests that HPV+ status is an important prognostic factor associated with a favourable outcome in head and neck cancers [36,40-43]. In a recent prospective multi-centre clinical study (as opposed to the retrospective studies listed above), data from Maura Gillison's lab demonstrated that patients with HPV+ tumours had better response rates after induction chemotherapy (82% vs. 55%), and after chemoradiation treatment (84% vs. 57%) compared to patients with HPV- tumours. Furthermore, they showed that after a median follow-up of 39.1 months, patients with HPV+ tumours had an improved overall survival of 33%, and after adjustment for age, tumour stage and ECOG status, a lower risk of progression and death from any cause, compared to those with HPV- tumours [44]. Although larger confirmatory studies are required, this study provided strong evidence that HPV+ tumour status was 1) associated with a better response to current treatment regimes, 2) associated with a much improved survival rate, and risk of progression, compared to HPV- tumour status.

The study above supports the available evidence that HPV+ tumours are a completely distinct epidemiological, biological and clinical subset of tumours from HPV- tumours [45]. Molecular evidence identifying differential gene expression between HPV+ and HPV- tumour groups support this line of thought [46-48]. Weinberger et al (2006) demonstrated 3 distinct classes of tumours based on HPV DNA detection and p16 detection [40]. They also proposed a biological model of HPV-induced head and neck cancer based on their findings (Class III). In this model, high-risk HPV E6 and E7 (predominantly HPV 16) proteins inactivate p53 and pRB, with subsequent upregulation of p16. Crucially, the model eliminates the need for mutational inactivation of p53 and pRB (leaving these tumour suppressor genes essentially intact). Tobacco and alcohol-related head and neck cancers would have mutations in p53 and pRB, deleting the activity of these pathways. The presence of intact p53 may therefore be one mechanism whereby removal of HPV E6 and E7 expression (for instance, by the immune response killing E6/E7 expressing cells) leads to the restoration of apoptotic pathways rendering the tumour more sensitive to chemoradiation treatment. The absence of "field cancerization" may be another factor leading to a better prognosis for HPV+ cancers. This term has been used to describe the presence of early genetic changes in epithelia from which multiple independent lesions can arise, and is typically seen in alcohol and tobacco -related tumours. This may also lead to the better treatment response profiles reported.

Finally, the better prognosis and treatment responses to chemotherapy and radiotherapy by HPV+ tumours may mean that HPV status detection is required to better plan and individualise patient treatment regimes.

### Current treatment options available for head and neck cancer

#### Treatment options for early stage disease

Approximately 30 to 40% of patients present with early stage I/II disease. These patients in general are treated with curative intent using either single modality treatments using either radiation or surgery alone. Because both modalities result in similar rates of local control and survival, the choice of treatment is usually based upon an assessment of functional outcomes and competing morbidities. When surgery is used as primary treatment, adjuvant radiotherapy is historically added if there are positive or close margins, bone erosion or pathologically positive lymphnodes, although combined radiochemotherapy has been shown to further improve outcome in some high risk patients [49].

Although early stage disease is associated with an excellent prognosis, these patients are still at high risk for recurrence and second primary tumours and need close monitoring.

### Potentially resectable local advanced disease

Treatment goals for patients with resectable locally advanced disease are to maximize cure while maintaining functional status through organ preservation. Historically surgery has been combined with postoperative radiotherapy, especially when risk factors, such as positive surgical margins, perineural or lymphovascular involvement, bone or cartilage invasion, T3/4 or nodal disease (N2-3) or extracapsular lymph node extension, were present. However with this approach locoregional control and 5-year survival was usually below 30%, and therefore combined modality approaches, which included chemotherapy, were tested in large randomized trials.

### Definitive Radiochemotherapy

A nonoperative approach is favored for patients in which surgery followed by either radiation alone or radiochemotherapy may lead to severe functional impairment. The feasibility of non-surgical approach in this situation was proven by the RTOG 9111 trial [50]. This study randomized 547 patients with stage III and IV supraglottic or glottic cancer into one of three arms: radiotherapy alone, concurrent radiochemotherapy with high dose-cisplatin or induction chemotherapy with cisplatin and fluorouracil followed by radiation. Preservation of the larynx and local control were best achieved with concurrent chemoradiotherapy, which led to an absolute reduction of 43% in the rate of laryngectomy. Larynectomy-free survival was better in both chemotherapy-containing arms compared to radiation alone, but overall survival was not different in the three treatment arms. This study established concurrent chemoradiotherapy as accepted standard for patients with advanced laryngeal cancer who want to preserve their larynx. However toxicity remains a significant problem with this approach, but recent advances in radiation such as the implementation of intensity-modulated radiation therapy or fractionation of treatment have been shown to reduce late toxicity without lowering local control rates or survival [51,52].

### Postoperative Chemoradiotherapy

Patients with oral cavity lesions or those with bone/cartilage invasion or gross organ destruction are usually clear candidates for initial surgery. To improve the limited survival rates in this patient population, two large phase 3 studies have been performed to determine whether the addition of cisplatin to radiotherapy improves the outcome, as compared with radiotherapy alone. Whereas in RTOG 9501 concurrent chemoradiotherapy only reduced the risk of loco-regional recurrence without differences in survival, in EORTC 22931 both progression-free survival and overall survival were significantly longer in patients receiving postoperative chemoradiotherapy compared to radiotherapy alone [49,53]. However, both studies reported a significant increase in toxicities, such as mucositis, bone marrow suppression and fibrosis, so that this aggressive treatment should be reserved for patients with good performance status and high-risk features, such as positive surgical margins or extracapsular extension. Patients with comorbity or low risk features should receive postoperative radiation alone.

### Induction chemotherapy

With combined modality treatment as described above local control rates have risen, but survival still remains poor, since distant metastasis have become a major site of fatal recurrence. Since systemic chemotherapy has been shown to decrease the risk of distant metastasis, several study groups tested induction chemotherapy followed by radiochemotherapy in patients with locally advanced HNSCC. A meta-analysis showed a 5% survival benefit for induction chemotherapy using cisplatin and fluorouracil [54]. Since docetaxel has shown substantial single-agent activity in the palliative setting, it was evaluated in the induction regimens in two large randomized multicenter trials. In the European TAX 323 trial, 358 patients with unresectable, locally advanced stage III and IV tumours received either docetaxel, cisplatin and fluorouracil or cisplatin and fluorouracil for four cycles followed by radiotherapy alone. Median overall survival (18.6 vs. 14.2 months) and progression-free survival was significantly longer for the patients who received the taxane-containing regimen [55]. These data were confirmed by the TAX 324 study, in which induction treatment was followed by concurrent radiochemotherapy instead of radiation alone. 501 patients were randomized to PF versus TPF and the responding patients received 7 weeks of concurrent chemoradiationtherapy with Carboplatin. The median survival was 70.6 month for the TPF group compared to 30.1 months in the PF group [56]. Because of these results TPF is the new standard for patients who are treated with induction chemotherapy, however the most important question whether induction chemotherapy followed by radiochemotherapy is better than radiochemotherapy alone remains open until results of ongoing trials addressing this problem are available.

### Recurrent and distant metastatic disease

Up to 50% of patients who die from HNSCC have locoregionally recurrent disease as the sole site of failure. Treatment options for those patients depend on primary treatment strategies. Patients treated with surgery alone should receive radiotherapy. Previously irradiated patients who have potentially resectable recurrence should undergo resection. If surgical salvage is not feasible alternatives include additional irradiation or palliative chemotherapy.

Although chemotherapy is of major palliative benefit in patients with symptomatic metastatic or incurable recurrent disease, impact on survival remains unclear. The average survival for patients receiving chemotherapy is six to eight months, which is only slightly more compared to supportive care alone. Therefore palliative treatment strategies should focus not only on response rates but also on toxicities and life quality aspects.

Among the most active and commonly used single agents are cisplatin, 5-FU and taxanes with response rates between 20 and 40%. Higher response rates can be reached by chemotherapy doublets, but no standard regimen could be established so far. Due to a better toxicity profile taxanes are promising candidates replacing the mostly used 5-FU as platin partner in the palliative setting, A randomized phase III study comparing cisplatin plus paclitaxel to cisplatin plus infusional 5-FU, showed that the taxan/platin combination was associated with less diarrhea, mucositis and myelosuppression and better life quality for patients [57]. For patients in excellent performance status chemotherapy triplets using a combination of platinum/taxane plus either 5-FU or ifsosfamide might be an option which is generally associated with higher response rates. However they are also more toxic and whether survival is better as compared to a doublet is not proven by a controlled randomized trial.

### Therapies targeting Epidermal Growth Factor Receptor

The epidermal growth factor receptor (EGFR), a member of the ErbB family, has been identified as therapeutic target for HNSCC and several other malignomas like colorectal adenocarcinomas and non-small cell lung cancer. Evaluation of EGFR-targeted therapies in HNSCC-patients was based on the observation that EGFR is highly expressed in many tumours and that EGFR overexpression was associated with reduced survival in several studies.

For clinical use EGFR can be targeted either by antibodies recognizing the ligand-binding domain of EGFR or by EGRF tyrosine kinase inhibitors, which interfere with ligand-binding induced signaling events. EGF has multifactorial effects hence unless extremely specific any aborogation of its activity may be associated with significant unanticipated deleterious side effects in addition to an anti-tumour activity hence e.g. associated cardiovascular co-morbidity with anti-EGFR antibody therapy must be appreciated.

### Anti-EGF-R antibody therapy

Cetuximab, a humanized mouse anti-EGF-R IgG1 monoclonal antibody, improved locoregional control and overall survival in combination with radiotherapy in locally advanced tumours [58]. In recurrent or metastatic disease resistant to platinum-based chemotherapy a randomized phase III study involving 442 subjects demonstrated a survival benefit for patients treated with cetuximab in addition to platinum-based therapy [59]. Median survival times were 10.1 months for the cetuximab plus chemotherapy versus 7.4 months for the study arm with chemotherapy alone. With the exception of skin rashes cetuximab did not add significant toxicities to the standard treatment, however similar to observations in colorectal cancer one study reported improved survival for patients who develop rashes in response to cetuximab. Of note, therapeutic response has not been found to be positively related to EGR-F protein levels, a phenomenon which is already known from other malignancies.

Other more humanized anti-EGF-R antibodies such a panitumumab or zalutumumab are currently being evaluated in phase II/III clinical trials and might evolve as alternatives to cetuximab in the future [60].

### EFFR-targeted TKIs

Two reversible competitive EGFR-selective TKIs have been evaluated in metastatic HNSCC with therapeutic response rates between 4 to 10%. Erlotinib which was first approved for treatment in lung cancer is currently being tested in several phase II trials in combination with gemcitabine or platinum-based chemotherapy. Preliminary response rates above 20% suggest significant efficacy. Gefitinib, a second TKI with proven efficacy in patients with specific subtypes of lung cancer, is currently under investigation in HNSCC. However phase III trials will be required to determine whether erlotinib or gefitinib improves survival compared to standard care.

HER 2 is another member of the ERB family which is elevated in a significant proportion of HNSCC. EGFR and HER 2 heterodimize to form functional signaling complexes, suggesting that TKIs, which are able to target both, EGFR and HER 2, may result in enhanced clinical responses [61]. Lapatinib, licensed for use in HER2-overexpressing breast cancers, is a dual-specificity, reversible TKI, which is presently evaluated in patients with recurrent or metastatic HNSCC. Unfortunately preliminary results show no significant improvement.

So far EGFR-targeted treatments show significant but limited response rates. This is likely due to activation of compensatory tumour-survival cell signaling pathways in response to TKI or antibody treatment. More laboratory research is needed to understand effects of the therapy as well as tumour compensatory changes. These changes are not expected to be uniform across HNSCC patients or tumours. However, a more detailed molecular characterization of signaling pathways in HNSCC tumours prior to therapy and alterations resulting from targeting therapies might help to define subpopulations of responsive patients and to define novel targets in the future.

## Summary

• The incidence of head and neck squamous cell carcinoma (HNSCC) has been gradually increasing over the last 3 decades.

• Human papillomavirus (HPV) induces a subset of head and neck carcinomas

• Oral HPV infection is likely to be sexually acquired.

• HPV+ status is an important prognostic factor associated with a favourable outcome.

• anti-EGFR targeted therapy improves locoregional control and overall survival in combination with radiotherapy in locally advanced tumours.

• Detailed molecular characterization of signaling pathways in HNSCC tumours prior to therapy might help to define subpopulations of responsive patients

## Competing interests

The authors declare that they have no competing interests.

## Authors' contributions

PG, MG, JE, HS performed the literature research and composed the manuscript.

MS, LS, TU, WJ and MBS critically revised the manuscript.
